# A new, enigmatic family for new genus and species of Polyneoptera from the Upper Permian of Russia

**DOI:** 10.3897/zookeys.130.1487

**Published:** 2011-09-24

**Authors:** Andrej V. Gorochov

**Affiliations:** Zoological Institute, Russian Academy of Sciences, St Petersburg 199034, Russia

**Keywords:** new taxa, possible Polyneoptera, Alexarasnia, Alexarasniidae, Russia, Upper Permian

## Abstract

Alexarasniidae **fam. n.** and *Alexarasnia rossica* **gen. et sp. n.** are described from the Upper Permian of European Russia. Systematic position of this enigmatic family within the infraclass Polyneoptera is unclear.

## Introduction

**Figures 1–2.**
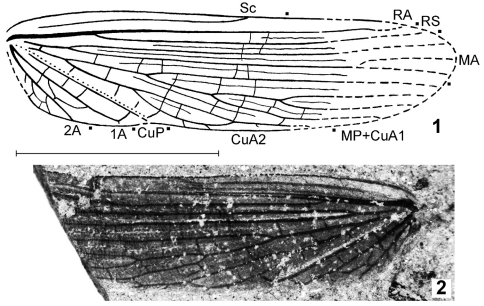
*Alexarasnia rossica* gen. et sp. n., tegmen, holotype PIN 3840/63 (part) **1** scheme of venation **2** photograph of fossil. Scale bar 5 mm.

Among numerous fossil insects collected from the Upper Permian locality Isady (European Russia), there are two wings with an unusual venation which does not allow me to include these fossils in any known order of the subclass Pterygota. One of these wings (Figs 1, 2) is not deformed and has the partly parallel longitudinal venation. Wings with such venation are aerodynamically unfit for flight, therefore parallel venation may be developed only in forewings which are used as tegmina or elytra for the protection of hind wings during rest. The main organs of flight in insects with such forewings are the hind wings, and all these insects belong to the order Coleoptera or to the infraclass Polyneoptera (= orthopteroid insects). The above-mentioned Upper Permian wings are with the well-developed venation including crossveins, i. e., they were clearly not as sclerotized or leathery as elytra in Coleoptera. So, it is most probable that these wings are tegmina belonging to Polyneoptera.

## Systematic Paleontology

### Family Alexarasniidae

Gorochov fam. n.

urn:lsid:zoobank.org:act:D6A2D4C4-6E69-4450-B12F-2488A6D9B6B7

http://species-id.net/wiki/Alexarasniidae

#### Type genus.

*Alexarasnia* gen. n.

#### Composition.

Only the genus *Alexarasnia* gen. n.

#### Diagnosis.

**Figures 3–4.**
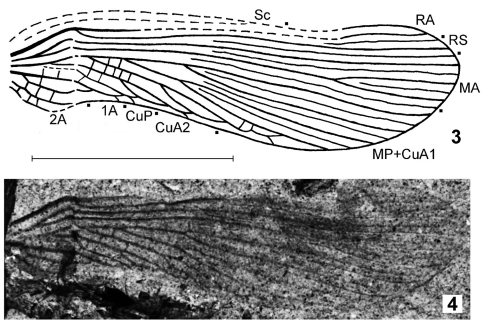
Alexarasniidae gen. et sp. indet., tegmen, specimen PIN 3840/1232 (part) **3** scheme of venation, **4** photograph of fossil. Scale bar 5 mm.

Tegmen (Figs 1–4) differs from that of Titanoptera, majority representatives of Orthoptera, and Paleozoic and Triassic Phasmatoptera in the absence of precostal area (presence of this area is a synapomorphy of all these orders). From the other representatives of two latter orders, this family differs in the reduction of Sc branches in the tegmen and/or clearly more numerous longitudinal tegminal veins (CuA has six or more branches in Alexarasniidae and four or less branches in all the other representatives of Phasmatoptera). Tegminal venation of the new family is distinguished from that of all the other orders of Polyneoptera by the partly parallel longitudinal veins in combination with the following characters: straight CuP, very narrow radial and interradial areas, and reduction of branches of RA and RS (from Dictyoptera); reduction of branches of Sc and/or RS, partial fusion of distal parts of some longitudinal veins with formation of long loop-like cells along anal edge of tegmen, and presence of intercalary veins between majority of longitudinal veins (from Grylloblattida as well as from the families Lemmatophoridae Sellards, 1909 and Atactophlebiidae Martynov, 1930 possibly belonging to the extinct order Eoblattida; Gorochov 2004); the latter characters as well as distal part of Sc not fused with RA (from Plecoptera and the other taxa of Eoblattida); tegmen not leathery with well-developed venation (from Dermaptera), and distinctly more numerous longitudinal veins (from Embioptera). Parallel venation of the tegmen is also present in the enigmatic family Chresmodidae Handlirsch, 1906 belonging to an unknown order of Polyneoptera (Delclòs et al 2008; Zhang et al 2008a, b); Alexarasniidae is distinguished from it by the distal half of tegminal CuP situated not parallel to the anal tegminal edge and more numerous branches of CuA in tegmen (6–9 instead of 3–4).

### Genus Alexarasnia

Gorochov gen. n.

urn:lsid:zoobank.org:act:E0774B66-F8F3-4BB8-AEF6-648EE189F4A6

http://species-id.net/wiki/Alexarasnia

#### Type species.

*Alexarasnia rossica* sp. n.

#### Description.

Tegmen (Figs 1, 2) moderately narrow, with parallel Sc, RA, RS, branches of MA and MP+CuA1, and short distal part of CuA2; all areas between longitudinal veins more or less narrow; majority of them with intercalary veins; Sc slightly concave, without branches and with apex situated near middle part of costal edge; R+RA slightly convex, but all longitudinal veins between this vein and CuP neutral; R and MA with proximal bifurcation situated in proximal part of tegmen; RA and RS probably simple (single); MA with only two branches in proximal and middle parts of tegmen; proximal part of MP before anastomosis with CuA1 indistinct; MP+CuA1 with four branches as minimum; CuA2 with two branches forming cell-like structure looking as a result of fusion of their distal parts; CuP concave (area near this vein with distinctly concave fold shown in Fig. 1a by dotted line), oblique and straight, but with small distal part probably looking as a result of its fusion with CuA2; 1A hardly convex, similar to CuP in shape; 2A neutral, looking as a single, oblique, and straight vein (its branches somewhat similar to crossveins); true crossveins simple and sparse.

#### Included species.

Only the type species.

#### Etymology.

The genus is named in honor of the famous Russian paleoentomologist Prof. Alexandr P. Rasnitsyn.

### Alexarasnia rossica

Gorochov sp. n.

urn:lsid:zoobank.org:act:5938A644-1656-4685-80B9-27278B946A4C

http://species-id.net/wiki/Alexarasnia_rossica

#### Holotype.

PIN (Paleontological Institute, Russian Academy of Sciences, Moscow) 3840/63, part and counterpart of imprint of tegmen (sex unknown) with indistinctly preserved distal area; Russia, Vologda Region, Velikij Ustyug District, left bank of Sukhona River near Isady Village (locality “Isady”); Upper Permian (Tatarian), Severodvinian Stage.

#### Description.

Tegmen (Figs 1, 2) with all veins more or less dark; membranes between them slightly darkened, but with somewhat lighter subcostal area; proximal bifurcation of MP+CuA1 and that of CuA2 situated rather far distad from proximal bifurcation of R. Length of tegmen 12 mm.

#### Etymology.

The specific epithet is adapted from “Rossia” (Latin for Russia).

## Discussion

Another tegmen from the same locality (PIN 3840/1232; Figs 3, 4) has the similar size, coloration of veins, and type of venation. However this fossil is deformed (with the middle part contracted in costal-anal direction), and as a possible result of this deformation, its venation looks less parallel than it might be prior to deformation. Moreover, its MP+CuA1 has six branches (probably more numerous than in holotype of *Alexarasnia rossica*), proximal bifurcation of MP+CuA1 situated near proximal bifurcation of R (compare with the description of *Alexarasnia rossica*, above), CuA2 shorter, and area between 1A and 2A remarkably wider (see Figs 1 and 3). These differences do not allow me to determine this wing more exactly, because it may belong to another sex of *Alexarasnia rossica*, to another species of *Alexarasnia*, or to another genus of Alexarasniidae.

Position of the new family within Polyneoptera is unclear. However, its tegminal venation looks more or less intermediate between the enigmatic Jurassic-Cretaceous water-striding family Chresmodidae [its very parallel tegminal venation was clearly figured by Delclòs et al (2008: Fig. 6); but in the same paper in figure 8, these authors figured the tegmen of Phasmatoptera from the superfamily Susumanioidea which was included mistakenly by them in Chresmodidae] and the Early and Middle Permian families Lemmatophoridae and Atactophlebiidae (possibly belonging to Eoblattida; Gorochov 2004). The mode of life of these Permian families was probably also connected with water (Carpenter 1935; Storozhenko 1998). In the latter publications, it was suggested that Lemmatophoridae (Carpenter 1935) and Atactophlebiidae (Storozhenko 1998) had a primitive postembryonal development, with an increase in the number of tarsal segments. This ancient type of development is absent now in all polyneopterans, but such instability of tarsal composition might be one of the prerequisites for the additional increase in the number of tarsal segments in Chresmodidae. Alexarasniidae (especially unknown body fossils) may prove to be useful in understanding this adaptive process.

## Supplementary Material

XML Treatment for Alexarasniidae

XML Treatment for Alexarasnia

XML Treatment for Alexarasnia rossica
